# Commentary: Case Report: Lenvatinib for the treatment of recurrent hepatocellular carcinoma in people living with HIV: a report of two cases

**DOI:** 10.3389/fonc.2024.1372745

**Published:** 2024-04-05

**Authors:** Cuiming Sun, Ying Wen, Jin Zhou

**Affiliations:** ^1^ The Second Department of Infectious Disease, The First Hospital of China Medical University, Shenyang, China; ^2^ Medical Oncology Department of Gastrointestinal Cancer, Liaoning Cancer Hospital Institute, Cancer Hospital of Dalian University of Technology, Shenyang, China

**Keywords:** lenvatinib, hepatocellular carcinoma, recurrence, people living with HIV (PLWH), adverse events (AEs) 1

## 1 Introduction

Although higher HCC incidence risk was reported among people living with HIV (PLWH) compared to general population, it actually declined from 2001 to 2019 (1), especially among cases with tenofovir (TDF) usage (2). However, poor adherence to HCC screening (3), delayed HCC diagnosis (4) and scarce data about tyrosine kinase inhibitors (TKIs) and immune checkpoint inhibitors (ICIs) (5) were observed among HIV/HBV or HIV/HCV patients.

The efficacy and tolerability of lenvatinib in treating HCC in PLWH were seldom reported. The recent publication of Morsica G et al, “Case Report: Lenvatinib for the treatment of recurrent HCC in people living with HIV: a report of two cases” firstly described the application of lenvatinib in two PLWH with unresectable HCC recurrence (6). We appreciated the authors for providing precious clinical experience. Whereas, there are several key issues deserved to be further addressed.

## 2 Commentary and discussion

Firstly, HIV-associated virological, immune status and integrase strand transfer inhibitors-containing anti-retroviral therapy (ART) regimens in these two reported cases did not cause negative impact on the efficacy, tolerability and drug-to-drug interactions of lenvatinib. Importantly, for these two cases, curative treatments for HCC with Barcelona Clinic Liver Cancer (BCLC) stage 0 were equally allocated according to HCC stage regardless of HIV status. HIV coinfection has no impact on the survival after diagnosis of HCC (7).

Secondly, lenvatinib related adverse events (AEs) in these two cases including uncontrolled hypertension and upper gastrointestinal bleeding should be discussed in detail. The common AEs of lenvatinib include hypertension, diarrhea, decreased appetite and weight, palmar-plantar erythrodysesthesia, and proteinuria, which usually occur in the first 1 to 2 months after lenvatinib treatment. In REFLECT trial, overall occurrence of hypertension in lenvatinib group was 42.2%, with grade≥ 3 hypertension occurring in 23.3% of patients, which was actually higher than sorafenib group (overall occurrence 30.3%, ≥ grade 3: 14.3%) (8). A total of ten real-world studies were literaturally reviewed (9), suggesting the tolerability and safety profile of lenvatinib were generally similar to that were seen in REFLECT trial. When detected early and managed appropriately, occurrence of hypertension did not impair the outcome of HCC patients treated with lenvatinib (10). Several studies showed in patients with liver transplantation (LT) receiving adjuvant lenvatinib treatment, incidence rate of hypertension was 42.9%-64.3% (11–13), consistent with the toxicity among non-LT HCC patients (8, 14–16). The available data about sorafenib treatment in PLWH showed its safety was similar to HIV-negative participants (17). Both lenvatinib and sorafenib are multikinase inhibitors, thus it is reasonable to speculate that the refractory hypertension was not ascribed to HIV infection in case 1.

HCC cases with cirrhosis increase the risk of bleeding from esophageal/gastric varices (EGV), which is a key concern in assessing the adoption of lenvatinib. The incidence of EGV bleeding was 3% in HCC patients treated with lenvatinib, with Child-Pugh B, portal vein thrombosis and platelet count <150,000/μl being the risk factors (18). It is necessary to perform gastroscopy to evaluate EGV status in these two cases before lenvatinib treatment because their platelet counts were <150,000/μl, though their Child-Pugh scores were level A. The level of albumin, prothrombin/international normalized ratio, and bilirubin in these two cases were relatively stable throughout 3-6 years of treatment, but liver function has limited value as a predictive biomarker for EVG. Other methods such as the new five-stage classification of liver cirrhosis might be better than Child-Pugh’s classification for EGV assessment (19). Furthermore, the stiffness measurement of the liver and spleen is also helpful in assessing portal hypertension or predicting variceal bleeding.

Thirdly, HCC progression was observed in case 1 after 6 months of lenvatinib, which suggested poor efficacy. In REFLECT trial, lenvatinib was shown to be non-inferior to sorafenib in overall survival in treating advanced HCC (8). In real-world studies, lenvatinib was superior to sorafenib in OS and PFS in patients with advanced HCC (20). The efficacy of lenvatinib in post-LT HCC recurrence patients was similar to non-LT HCC patients (12). The Blood concentration of FK506 was not influenced by lenvatinib (13). However, to decrease the risk for HCC recurrence post-LT, reducing the calcineurin inhibitors dosage, and combining or completely switching to the mammalian target of rapamycin inhibitors are the preferred immunosuppressor regimens (21). Late peritoneal metastasis with HCC recurrence (beyond 2 years post-transplant) happens sporadically, and peritoneal metastasectomy (without metastasis in other organs) or combining sorafenib was an effective therapy (22). HIV-positive or negative patients undergoing LT for HCC have comparable post-LT survival, while microvascular invasion, HCC diameter, and number of HCC nodules were predictors of recurrence post-LT (23). So far, radiofrequency ablation has no significant differences in OS between HIV-positive and HIV-negative patients (24).

Lastly, HCC survival prediction should be discussed. BCLC stage, decompensated cirrhosis, alpha-fetoprotein level, age, and radiological aggressiveness are associated with death. For cases with unresectable BCLC stage B HCC recurrence, noncurative treatments include trans-catheter arterial chemoembolization (TACE), trans-catheter arterial radioembolization, TKIs, and supportive care. The combination (sequential or alternating) of lenvatinib and TACE (even followed by conversion surgery), or regorafenib could be the beneficial therapeutic strategy (25). ICIs-based treatment should be considered as an alternative treatment after lenvatinib discontinuation. ICIs have been also evaluated in PLWH with advanced cancers including some HCC cases (26–28). When HCC progressed after lenvatinib treatment, regorafenib monotherapy (29) or regorafenib combined with PD-1 inhibitor (30), or atezolizumab plus bevacizumab (31), or nivolumab plus ipilimumab (32), could be the alternative options as the second-line therapy. However, for post-LT individuals, rejection incidence of ICIs and bleeding risk of antivascular targeted agents should be carefully assessed.

In conclusion, the efficacy and safety of lenvatinib in treating PLWH with HCC should be evidenced by increasing case reports and clinical trials. HCC treatment should be equally recommended in HIV-positive and HIV-negative individuals. Implementing HBV vaccination, adopting TDF-containing ART regimen, improving direct-acting-antivirals application, strengthening HCC surveillance and building a multidisciplinary team for PLWH are helpful in decreasing HCC incidence in PLWH, diagnosing HCC at earlier stages, and detecting HCC recurrence timely.

## Author contributions

CS: Writing – original draft, Writing – review & editing. YW: Conceptualization, Supervision, Writing – original draft, Writing – review & editing. JZ: Conceptualization, Investigation, Writing – original draft, Writing – review & editing.
